# Editorial: Psychological Mechanisms That Affect Economic Decisions to Work Longer

**DOI:** 10.3389/fpsyg.2019.03003

**Published:** 2020-01-22

**Authors:** Gabriela Topa, Joanne K. Earl, Jacquelyn B. James

**Affiliations:** ^1^Department of Social and Organizational Psychology, National University of Distance Education, Madrid, Spain; ^2^Department of Psychology, Macquarie University, Sydney, NSW, Australia; ^3^School of Social Work, Boston College, Chestnut Hill, MA, United States

**Keywords:** retirement, older workers, work ability, retirement preferences, age diversity, age-diversity practices, self-employment, time perspective

Progressive aging of the Baby Boom generation, early workplace withdrawals and international trends toward longevity place increasing pressure on governments to provide economic solutions. One solution is to promote financial self-sufficiency and shift responsibility from governments to individuals. This involves, in part, abolishing mandatory retirement ages and devising strategies to keep people at work beyond conventional retirement ages. Working longer, when one wants to and can, has been shown to provide psychological benefits such as the ability to remain socially and intellectually connected.

There are also obvious economic benefits, especially for disadvantaged groups such as women, immigrants, and low-wage workers who may be ill-equipped to retire comfortably. There are, however, two sets of competing demands, i.e., balancing the desire and economic demand to work longer within current contemporary workplace designs and the cognitive, physical, and psychological capacities of workers to fulfill these demands.

Scholarship on the psychological mechanisms that underlie economic behavior is crucial to our understanding of how bridge employment opportunities, flexible work options, training for new careers, and starting new businesses might contribute to higher proportions of older workers remaining in the labor market. The expansion of such options is believed to enhance both employer and individual responses to the changing context of aging and work.

Thus, the *psychological mechanisms that affect economic decisions to work longer* was the focus of our special issue. We sought diverse perspectives from around the world illuminating the opportunities and challenges of older workers. We succeeded in securing 11 papers from 14 countries: Australia, Belgium, Brazil, Chile, China, Ecuador, Germany, Italy, Japan, Netherlands, Portugal, Spain, Switzerland, and the United Kingdom. Three themes emerged that potentially inform employment, human resources, and educational practices; customization and adaptation to the workplace; understanding individual differences; and promoting self-insight to improve planning behavior.

Four papers by Yeves et al., Climent-Rodríguez et al., Peters et al., and Sousa et al. point to opportunities within organizations to introduce human resource practices that support older workers. Multi-faceted and complex factors determine the response to working longer and particularly the importance of employability. Yeves et al. investigate the relationship between perceived employability and age on job insecurity and job satisfaction. They find that age plays an important role among employees with high, but not low, perceived employability. Perceived employability by older workers provides little protection from the threat of losing one's job.

Similarly, Climent-Rodríguez et al. show that losing a job may mean different things across the career cycle, with more pronounced effects at older ages. When older people lose their jobs, they feel it more intensely and over a longer period than do their younger counterparts. Put simply the longer one is out of work, the less a person believes in his or her employability.

Other people's reactions to older workers, along with employment practices and again, perceived employability, matter too. In the paper by Peters et al., a Sustainable Human Resource Management framework is used along with Sustainable Career Development (SCD) to investigate the relationship between age, self-reported employability, and stereotypes relating to older workers' productivity, reliability, and personal adaptability. Negative age-based stereotypes impact self-reported employability, exaggerating the effects of age.

Sousa et al. demonstrate the need for age diverse practices in the workplace that consider age-related changes in skills, preferences, and goals. The authors show that work engagement mediates the association between age-diversity practices and the preference for early or late retirement, and that this is particularly important for those with low work ability.

Together the papers in our first theme inform employment and human resource practices. Specifically, demonstrating the significance of age for employees with high perceived employability, the larger effects of losing one's job at an older age and the impact of negative aged-based stereotypes. Practical implications for organizations include the need for age diverse practices and management, concentrating on the intrinsic factors of older workers, opposing negative age-based stereotyping, creating an inclusive environment, and encouraging career opportunities at all ages.

The second theme encompassing three papers by Hu et al., Caines et al., and Molero et al. emphasize customization of roles and adaption to work environments through job crafting, being self-employed, and managing burnout to maximize working life. Papers by Hu et al., Caines et al., and Molero et al. point to opportunities for employees to interact differently with their environments, to fulfill long-held aspirations and introduce protective factors that make it possible to work longer. The opportunity to design a role that optimizes a person's interests and strengths is intuitively appealing to all workers but particularly older workers who may have a keener sense of what these are. Hu et al. link job crafting behaviors with job-related resources and work engagement. Self-employment is another possible solution for older workers wanting to explore job-crafting beyond traditional organizational boundaries and extend their working life. Caines et al. explore how attitudes to aging, one's future time perspective and perceived support from valued others predicts self-efficacy when opting for self-employment. The longer people think they will live the more likely it is that they will take up self-employment. How well-supported they feel by others to become an entrepreneur helps to determine their own perceived likelihood of success. Even when taking job crafting, self-employment and a strong desire to work for as long as possible into consideration, some occupations are just more susceptible to burnout than others. This is particularly true in those roles with high workloads and emotional labor demands, such as nursing. Strategies to stay at work for longer by nurses susceptible to burn out is the focus of a paper by Molero et al. The authors investigate the relationship between self-efficacy, self-esteem, and burnout. Self-care focusing on improving self-efficacy and self-esteem may provide an insulating effect for jobs with high workloads that otherwise result in burnout.

The papers in our second theme highlight the importance of understanding opportunities for customization of the work environment and adaptation necessary to enable a long, successful work life. Practical implications of these findings involve the need for promoting multilevel job crafting to improve job engagement and tailoring programs to help explore and evaluate self-employment opportunities. Organizations would benefit from developing and creating opportunities for older workers to explore ways of adapting jobs and to consider different ways of working together to maximize working life, satisfaction and well-being.

The third theme includes four papers by Noone et al., Schuabb et al., Topa et al. and Topa and Zacher that explore individual factors accounting for differences in planning behavior, the importance of insight and delivery of interventions at just the right time. Noone et al. emphasizes the need to plan for financial security further ahead to expand options and identifies individual factors that may account for differences in staying at work for longer. Push-pull factors are explored that keep people at work—balancing the individual demographic factors with aspects of work relating to fit and identity. The role of gender, financial pressure, physical health, and caregiving alongside three newer emerging factors: work-life conflict, work centrality, and person-job fit are explored. These factors along with physical health and caregiving are related to on-going workforce participation. Important differences emerge according to gender—caregiving was a reason for men to stay employed whilst for women it was person job fit. Limited financial pressure gave people reporting work-life conflict the option to leave work earlier than those reporting financial pressure.

One other factor associated with the ability to plan ahead is Future Time Perspective. Providing greater insight into Future Time Perspective (FTP) is the focus of the paper by Topa and Zacher who validate the use of a more specific measure of FTP, the Occupational Future Time Perspective in Spain with a local sample. The OFTP has three sub-scales relevant to aging research: perceived remaining time; focus on opportunities; and focus on limitations. Their validation of the measure provides new cohorts with a better understanding of their own behavior while paving the way for researchers to conduct cross-cultural research.

Two of our papers by Schuabb et al., and Topa et al. focus on the timing of skill acquisition in financial money management. Schuabb et al. consider influences on retirement savings and the important roles played by parental influence, retirement goal clarity and retirement planning activity level. Having clear goals about savings is an important intermediator between parental influence and retirement saving, while the amount of retirement saving was determined by retirement activity. Goal clarity imparted by parents from childhood may be necessary to instill values related to long-term planning.

Topa et al. point also to the importance of having a longer-term strategy around saving and management of finances. Their study with younger adults revealed the importance of combining investment advice and cognitive closure (i.e., coming to a clear and decisive opinion) to promote better financial management behavior, in relation to both the urgency of getting knowledge and the permanence of such knowledge.

This third set of papers informs educational practices, emphasizing the requirement for earlier financial security planning, retirement goal clarity, and consideration of parental influence to encourage earlier long-term planning and a smoother retirement transition. On an individual level, providing opportunities for reflection and self-insight might prompt better saving behaviors necessary for long-term financial security, and metacognitive strategies could be taught together with investment advice to improve financial decision making.

We hope that the 11 papers selected for our special edition expand our understanding of factors involved in decisions to work longer and inspire additional research to improve the lives of older people at work.

## Author Contributions

All authors listed have made a substantial, direct and intellectual contribution to the work, and approved it for publication.

### Conflict of Interest

The authors declare that the research was conducted in the absence of any commercial or financial relationships that could be construed as a potential conflict of interest.

